# Hair Regenerative Mechanisms of Red Ginseng Oil and Its Major Components in the Testosterone-Induced Delay of Anagen Entry in C57BL/6 Mice

**DOI:** 10.3390/molecules22091505

**Published:** 2017-09-08

**Authors:** Van-Long Truong, Min Ji Bak, Changook Lee, Mira Jun, Woo-Sik Jeong

**Affiliations:** 1 Department of Food and Life Sciences, College of BNIT, Inje University, Gimhae 50834, Korea; truonglongpro@gmail.com (V.-L.T.); bakmj0130@gmail.com (M.J.B.); 2 Department of Chemical Biology, Susan Lehman Cullman Laboratory for Cancer Research, Ernest Mario School of Pharmacy, Rutgers, The State University of New Jersey, Piscataway, NJ 08854, USA; 3 Department of Pharmaceutics, College of Pharmacy, Inje University, Gimhae 50834, Korea; lck1764@naver.com; 4 Department of Food Science and Nutrition, Dong-A University, Busan 49315, Korea; mjun@dau.ac.kr

**Keywords:** androgenic alopecia, hair re-growth, red ginseng oil, Wnt/β-catenin pathway, Shh/Gli pathway, linoleic acid, sitosterol

## Abstract

Hair loss (alopecia) is a universal problem for numerous people in the world. The present study was conducted to investigate the effects of red ginseng oil (RGO) and its major components on hair re-growth using testosterone (TES)-induced delay of anagen entry in C57BL/6 mice and their mechanisms of action. Seven-week-old C57BL/6 mice were daily treated with TES for 1 h prior to topical application of 10% RGO, 1% linoleic acid (LA), 1% β-sitosterol (SITOS), or 1% bicyclo(10.1.0)tridec-1-ene (BICYCLO) once a day for 28 days. Hair regenerative capacity was significantly restored by treatment of RGO and its major compounds in the TES-treated mice. Histological analysis showed that RGO along with LA and SITOS but not BICYCLO promoted hair growth through early inducing anagen phase that was delayed by TES in mice. Treatment of mice with RGO, LA, or SITOS up-regulated Wnt/β-catenin and Shh/Gli pathways-mediated expression of genes such as β-catenin, Lef-1, Sonic hedgehog, Smoothened, Gli-1, Cyclin D1, and Cyclin E in the TES-treated mice. In addition, RGO and its major components reduced the protein level of TGF-β but enhanced the expression of anti-apoptotic protein Bcl-2. These results suggest that RGO is a potent novel therapeutic natural product for treatment of androgenic alopecia possibly through hair re-growth activity of its major components such as LA and SITOS.

## 1. Introduction

Hair loss that refers to loss of some or all hairs on the head or body, which is caused by a wide range of factors from environments to genetics. Recently, the occurrence of hair loss has dramatically increased. Although hair loss is not a mortal disease, a common and emotionally distressing problem which affects social communication, mental health, and eventually overall quality of life.

Androgenic alopecia (AGA) is the most common case of hair loss, affecting both men and women at different ages [1,2]. AGA is characterized by abnormal alterations in hair cycle and hair follicle structure. Androgens are believed to shorten anagen phase, leading to an increasing number of hair follicles in the catagen and telogen phase, and delaying telogen-to-anagen transition [3]. The follicular miniaturization by androgens results in transformation of terminal follicles to vellus-like follicles, which produces thinner and shorter hairs [4]. AGA is associated to increased level of dihydrotestosterone (DHT) that is generated by enzymatic transformation of testosterone (TES) under the catalysis of 5α-reductase (5αR). Basically, DHT binds to the androgenic receptor (AR) to form hormone-receptor complex and then induces target genes associated to shortening the duration of the anagen, enhancing apoptosis of hair cells, miniaturizing the hair follicle, and finally hair loss [2]. TES also exhibits affinity with AR, however, its affinity is weaker than that of DHT [4]. The increased levels of 5αR and AR are found in patients with AGA [5].

Finasteride (FINAS), approved by US Food and Drug Administration, is widely used as an oral drug in many countries for treatment of androgen-related hair disorders such as AGA through its inhibition of 5αR activity [6]. However, FINAS exhibits highly variable efficacy between individuals and undesirable side-effects [7]. Therefore, alternative strategies for hair loss treatment are necessary and novel natural compounds have been attracting a great interest owing to their potential effectiveness, less side effects, and easy availability. Recently, serval natural compounds have been indicated to be potent therapeutic agents for treatment of androgenic hair loss. Water soluble red ginseng extract and its constituent ginsenoside-Ro have been demonstrated to enhance hair re-growth in the TES-induced AGA model through the their inhibitory effect on 5αR activity [8]. Wang et al. have shown that topical applications of white wax and policosanol from white wax promote hair growth in a mouse model of androgenic hair loss via inhibiting 5αR activity and increasing dermal papilla cell proliferation [9].

Ginseng (*Panax ginseng* Mayer), a long time traditional medicine in Korea, has been demonstrated to possess hair growth promoting activity [10,11]. Previously, we reported a variety of pharmacological activities of red ginseng oil, which was extracted from red ginseng using a supercritical CO_2_ fluid extraction system, such as antioxidant, hepatoprotective [12], and anti-inflammatory properties [13]. RGO has also been demonstrated to exhibit chemopreventive property through the induction of nuclear factor erythroid 2-related factor 2 (Nrf2)/antioxidant response element (ARE)-mediated cytoprotective genes [14]. Recently, chemical composition of RGO was revealed by GC/MS analysis, indicating that linoleic acid (LA), β-sitosterol (SITOS), and bicyclo(10.1.0)tridec-1-ene (BICYCLO) were three major components, occupying about 71% of RGO [14]. Linoleic acid, one of lipophilic constituents of *Lygodii Spora*, exhibits suppressing effect against 5αR activity and thereby contributing to hair re-growth activity of the plant’s ethanol extract in TES-treated C57Black/6CrSlc mice [15]. Linoleic acid from rice bran oil has also been reported to initiate anagen phase and exhibit hair growth promoting potential through inducing growth factors such as vascular endothelial growth factor (VEGF), insulin-like growth factor-1 (IGF-1), and keratinocyte growth factor (KGF), as well as through suppressing transforming growth factor-β (TGF-β) [16]. Additionally, β-sitosterol from plants has been shown to act as an inhibitor of 5αR activity, implying hair growth promoting capacity of β-sitosterol [17]. However, there is no report available so far on hair re-growth promoting activity of RGO and its components. Therefore, the present study focused on investigating hair regenerative effects of RGO and its major components including LA, SITOS, and BICYCLO in a mouse model of TES-induced AGA. In addition, the molecular mechanisms of RGO and its major compounds on the hair re-growth promotion were examined.

## 2. Results and Discussion

### 2.1. Hair Regenerative Effects of RGO and Its Major Components in the TES-Treated C57BL/6 Mice

**Figure 1:**
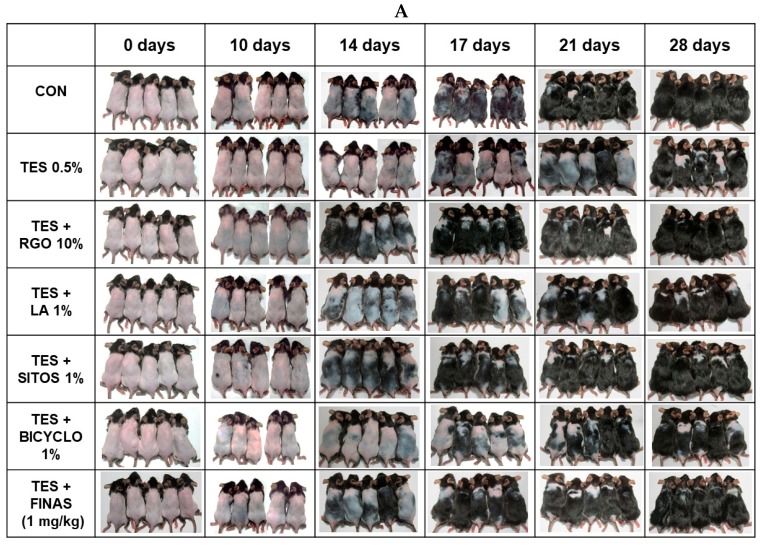

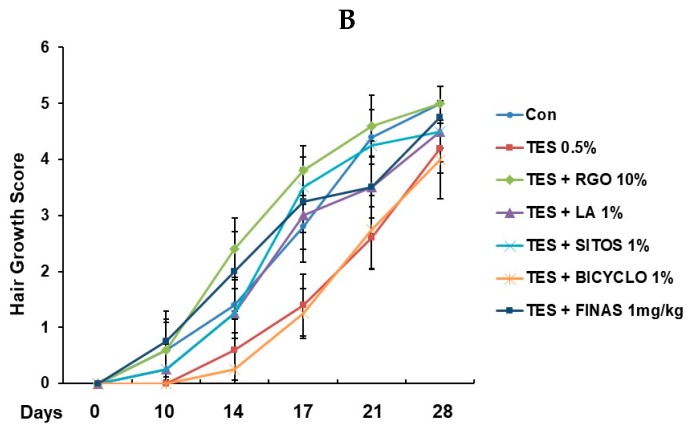
Hair regenerative effects of red ginseng oil (RGO) and its major compounds in the testosterone (TES)-induced androgenic alopecia C57BL/6 mouse models. The shaved-back skins of mice were daily treated with 0.5% TES for 1 h prior to topical application of 10% RGO, 1% LA, 1% SITOS, or 1% BICYCLO once a day for 28 days. Mice in the positive control group were treated with 0.5% TES and orally administered with FINAS (1 mg/kg) once a day for 28 days. The dorsal skins were photographed at day 0, 10, 14, 17, 21, and 28 (**A**). Hair re-growth efficacy score was measured as 0, 1, 2, 3, 4, and 5 in correspondence to 0%, 0–20%, 20–40%, 40–60%, 60–80%, and 80–100% of hair growth (**B**).

To evaluate hair regenerative activities of RGO and its major components in the TES-induced AGA models, telogenic dorsal skins of C57BL/6 mice were topically treated with RGO, LA, SITOS, or BICYCLO in the presence of TES once a day for 28 days. C57BL/6 mouse dorsal hair is known to have a time-synchronized hair growth cycle. The skin pigmentation is taken as an evidence for hair growth, which is bright pink in the telogen phase and turns into grey/black in the anagen phase. As shown in Figure 1, TES treatment apparently delayed the hair regeneration in mice. The control group exhibited grey skin at day 14, and short hair shafts at day 17 after depilation, and their dorsal skins were completely covered by visible hair shafts at day 28. The TES group remained the large areas of skin without pigmentation until day 17. At day 28, about 70–80% areas of the black skin of this group appeared with hair shafts. However, topical application of RGO, or its major compounds, restored hair regenerative capacity in the TES-challenged AGA mouse model. RGO-treated mice exhibited early visible hair shafts at day 14, while mice treated with LA or SITOS, two major compounds in RGO, expressed dark grey skin by 14 days and hair shafts appeared at day 17. On the other hand, BICYCLO treatment, another main component of RGO, was unlikely to promote hair re-growth in the TES-treated mice, which remained with less pigmentation until day 17 and contained large areas of skin without hair shafts after 28 days. The positive control FINAS, a well-known drug for treatment of androgenic alopecia, was confirmed to produce hair growth promoting effect on TES-induced C57BL/6 mice. Among all treatments, RGO seemed to produce the greatest effect on hair regeneration in the TES-treated mice and its effect was even greater than that of the drug FINAS. Recently, several natural products have been studied as potential agents for prevention and/or treatment of androgenic hair loss. Topical application of epigallocatechin-3-gallate provoked hair re-growth in the TES-induced androgenic alopecia B6CBAF1/j mice [18]. Extract of *Thujae occidentalis* semen reduced the degree of alopecia in a androchronogenetic alopecia B6CBAF1/j mouse model through inhibiting activity of 5α-reductase type 2 [19]. Topical application of ethanolic, petroleum ether extract, or isolate of petroleum ether extract of *Cuscuta reflexa* also increased the number of hair follicles, as well as the follicle anagen/telogen (A/T) ratio, thereby enhancing hair growth in the TES-treated rats [20]. Similarly, petroleum ether extract of *Citrullus colocynthis* Schrad fruits [21] and ethanol extract of *Adiantum Capillus veneris* Linm [22] have been found to exhibit hair re-growth promoting activity in animal models of TES-induced alopecia.

In the present study, we report, for the first time, the hair regenerative activity of RGO in the AGA model, and two major compounds including LA and SITOS seem to importantly contribute to the hair growth promoting activity of RGO.

### 2.2. Effects of RGO and Its Major Components on Hair Follicle Growth in the TES-Treated C57BL/6 Mice

**Figure 2:**
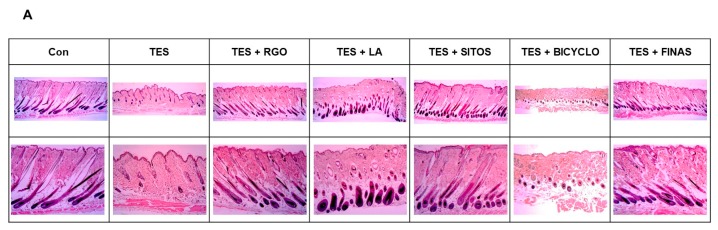

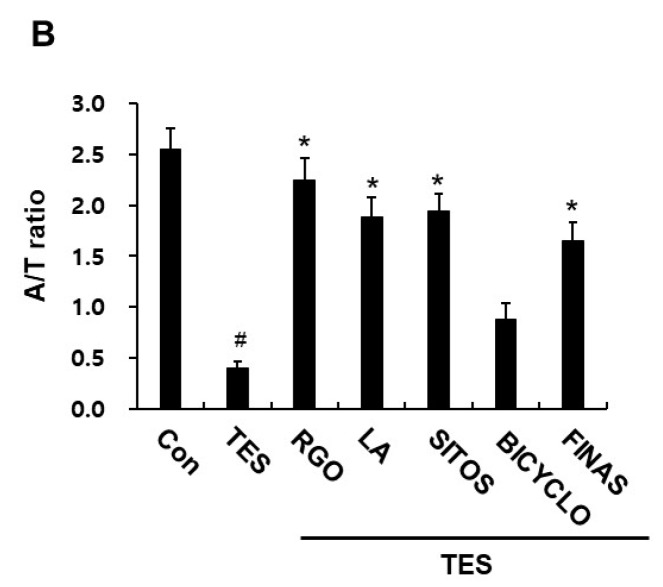
Effects of RGO and its major components on hair follicle development in the TES-induced androgenic alopecia C57BL/6 mouse models. The shaved-back skins of mice were treated with 0.5% TES for 1 h prior to topical application of 10% RGO, 1% LA, 1% SITOS, or 1% BICYCLO once a day for 28 days. Mice in the positive control group were treated with 0.5% TES and orally administered with FINAS (1 mg/kg) once a day for 28 days. The dorsal skin tissues of each group were collected at day 17 after treatment and subjected to hematoxylin and eosin (H&E) staining. (**A**) Representative photomicrographs of longitudinal sections are shown. Magnification ×40 (upper panel) and ×100 (lower panel); (**B**) Hair growth pattern (anagen/telogen ratio) in C57BL/6 mice. Data are presented as the mean ± SD of three independent experiments. ^#^ *p* < 0.05 significant difference as compared with vehicle-treated control group. * *p* < 0.05 significant difference as compared with TES-treated group.

To investigate whether RGO and its major components can accelerate hair re-growth and activate anagen phase in the TES-treated mice, dorsal skins from each group were collected after 17 days of depilation and subjected to H&E staining. Histological analysis showed that TES treatment obviously delayed the entry of anagen phase of hair follicles in the telogenic C57BL/6 mice compared with vehicle treatment, thereby inhibiting hair growth (Figure 2A). After 17 days of depilation, just a few hair follicles were formed in the dermis and entered late anagen II and early anagen III in the TES-treated group, whereas most of hair follicles appeared in deep subcutis and entered anagen V/VI in the control group. Topical application of RGO restored the progression of telogen-to-anagen transition of hair follicles, which was in anagen V/VI with hair shafts erupting out of epidermis in the TES-treated mice. Similarly, LA and SITOS treatments also converted the telogenic hair follicles to anagenic hair follicles that appeared in anagen VI/V. However, BICYCLO was unlikely to significantly promote telogen-to-anagen transition of hair follicles in the mouse model. Similar to RGO, the development of hair follicles in the FINAS-treated group was more apparent than that in the TES-treated group. In addition, the anagen/telogen (A/T) ratio in the TES group decreased compared with that in the control group (Figure 2B). Treatment with RGO, LA, or SITOS increased A/T ratio in the TES-treated mice, while treatment with BICYCLO slightly evaluated A/T ratio in TES-treated mice. The A/T ratio in the FINAS-treated group was also higher than that in the TES-treated group. These results suggest that RGO and its major components LA and SITOS induce premature telogen-toanagen transition of hair follicles, thereby promoting hair re-growth in the TES-treated mice.

### 2.3. Effect of RGO and Its Major Components on Expression of TGF-β, and Bcl-2 Proteins in the TES-Treated C57BL/6 Mice

**Figure 3:**
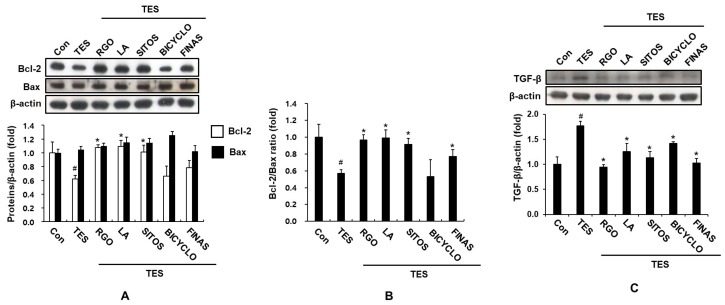
Effects of RGO and its major components on the protein levels of and Bcl-2 and Bax (**A**); Bcl-2/Bax ratio (**B**); and protein expression of TGF-β (**C**) in the TES-induced androgenic alopecia C57BL/6 mouse models. Dorsal skin tissues of each group were collected after 17 days of depilation. Protein expression levels of Bcl-2, Bax, and TGF-β in the mouse skin tissues were detected by western blotting. The intensity of total amount of each band was densitometrically measured and normalized against β-actin. Data are presented as the mean ± SD of three independent experiments. ^#^ *p* < 0.05 significant difference as compared with vehicle-treated control group. * *p* < 0.05 significant difference as compared with TES-treated group.

Hair cycle and regeneration are complex processes related to proliferation, differentiation, and apoptosis of hair cells. Apoptosis is one of the most important factors affecting cell survival as well as hair regeneration [23]. The members of Bcl-2 family including anti-apoptotic Bcl-2 and pro-apoptotic Bax proteins appear to act as the apoptosis control machinery of most cell types as well as regulation of hair cycle [24]. A previous study has indicated that cisplatin treatment enhances formation of hydroxyl radical and parallely decreases the Bcl-2/Bax ratio, resulting in apoptotic cell death of hair dermal papilla cells and keratinocytes, as well as massive hair loss [25]; whereas EGCG [26] and FINAS [27] increase the Bcl-2/Bax ratio, thereby enhancing dermal papilla cell survival and hair growth. In contrast, 6-gingerol exhibits suppressing effect on human hair growth and causes prolongation of telogen phase in mice possibly through decreasing the Bcl-2/Bax ratio and increasing apoptotic cell death of dermal papilla cells [28]. In addition, apoptosis in hair follicles is thought to play a crucial role in the process of androgenic hair loss [29]. It has been demonstrated that treatment of TES and DHT down-regulates Bcl-2 expression and promotes apoptosis in human dermal papilla cells [30]. Thus, we examined whether RGO and its major components could regulate the protein expression of apoptosis-associated genes including Bcl-2 and Bax. As displayed in Figure 3A, the expression level of anti-apoptotic protein Bcl-2 was attenuated in the TES-treated mice compared to the normal control after 17 days of depilation, suggesting increased hair cell apoptosis and prolonged telogen phase by TES. Treatment of RGO, LA, and SITOS, but not BICYCLO significantly restored the protein expression of Bcl-2 in the TES-treated mice and their restoration ability was even greater than FINAS treatment. On other hand, RGO and its major components did not produce a significant alteration in protein level of Bax in the TES-treated mice. Accordingly, the Bcl-2/Bax ratio significantly increased in the RGO-, LA-, SITOS-, and FINAS-treated mice compared to the TES-treated mice (Figure 3B).

In addition, TGF-β importantly contributes to the initiation of apoptotic cell death, inhibition of matrix cell growth, induction of catagen-like transition and hair loss [31,32]. TGF-β1 injection leads to premature catagen transformation through decreased follicular proliferation and increased apoptosis, whereas TGF-β1-deficient mice prolong the anagen phase [33]. Moreover, several studies have demonstrated that androgens up-regulate TGF-β in dermal papilla cells in bald scalp [34,35]. Ginsenoside-Re promotes cyclic growth of hair follicles and hair growth through suppressing TGF-β signaling pathway [36]. Therefore, inhibition of TGF-β might be a potential strategy for promoting hair growth. As an example, ginsenoside F2 has been reported to reduce apoptosis and TGF-β expression in DHT-treated hair cells and in DHT-induced hair loss mouse model, thereby promoting hair re-growth [37]. Similar to these results, in the present study, the mice treated with TES resulted in a remarkable increase in the protein level of TGF-β in skin tissues when compared with vehicle-treated control mice (Figure 3C). Conversely, the increased level of TGF-β by TES in dermal skin was down-regulated by topical application of RGO, as well as its major components including LA, SITOS, and BICYCLO. FINAS treatment also attenuated the expression of TGF-β in the TES-treated mice. These data suggest that RGO and its major components inhibit apoptotic pathways, leading to premature initiation of anagen, prolongation of anagen phase, and promotion of hair re-growth in the TES-induced delay of anagen entry in mice.

### 2.4. Effects of RGO and Its Major Components on Wnt/β-Catenin and Sonic Hedgehog (Shh)/Gli Pathways in the TES-Treated C57BL/6 Mice

**Figure 4:**
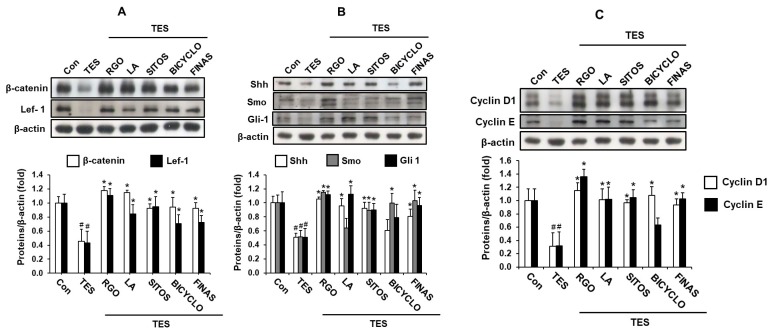
Effects of RGO and its major components on Wnt/β-catenin and Shh/Gli signaling pathways in the TES-induced androgenic alopecia C57BL/6 mouse models. Dorsal skin tissues of each group were collected after 17 days of depilation. Protein expression levels of (**A**) β-catenin and Lef-1; (**B**) Shh, Smo, and Gli-1; (**C**) Cyclin D1 and Cyclin E in the mouse skin tissues were detected by western blotting. The intensity of total amount of each band was densitometrically measured and normalized against β-actin. Data are presented as the mean ± SD of three independent experiments. ^#^ *p* < 0.05 significant difference as compared with vehicle-treated control group. * *p* < 0.05 significant difference as compared with TES-treated group.

Telogen-to-anagen transformation of hair follicles is an interaction between mesenchymal and epithelial cells mediated by various signaling pathways [38]. Wnt/β-catenin and Shh/Gli pathways play an important role in hair growth through the inducing of the telogen-to-anagen transition as well as maintaining the anagen phase of the hair cycle. In order to further investigate hair regenerative mechanisms of RGO and its major compounds, the protein expression of Wnt/β-catenin and Shh/Gli pathways-related genes in the TES-treated mice was examined. As shown in Figure 4A, TES produced the inhibitory effect on the protein expression of β-catenin and Lef-1 in the mouse skin tissues when compared with vehicle treatment only. However, mice treated with RGO as well as its main compounds such as LA, SITOS, and BICYCLO remarkably restored protein levels of Wnt signaling-related genes including β-catenin and Lef-1 in the TES-treated mice. The inducing effect of RGO on the expression of these proteins was greater than that of FINAS. Therefore, premature induction of anagen of the hair cycle and hair growth promotion by RGO and its major components including LA and SITOS might be mediated through activating the Wnt/β-catenin signaling pathway in the TES-treated mice.

The Wnt/β-catenin pathway is also an important signaling mechanism contributing to the initiation, development, and growth of hair follicles [39,40]. Activation of the Wnt/β-catenin pathway leads to enhanced expression of β-catenin-mediated genes essential for proliferation, growth, and differentiation of dermal papilla cells and around cells, which take part in increasing size and number of hair follicles, thereby promoting hair follicle regeneration and hair growth [41,42]. In addition, the Wnt/β-catenin signaling pathway plays a vital role in maintaining and prolonging the anagen phase. The duration of the anagen phase determines the length of the hair shaft [3,43]. Ablation of β-catenin results in the premature induction of the catagen phase of the hair cycle, implying that activation of β-catenin is required for maintaining the anagen phase of the hair cycle [44]. Furthermore, it has been demonstrated that androgen treatment inhibits the Wnt/β-catenin signaling pathway possibly through decreasing β-catenin protein and up-regulating the activity of glycogen synthase kinase 3β in dermal papilla cells, thereby resulting in androgenic alopecia [45,46]. The interaction of androgenic receptor with β-catenin in a androgen-dependent manner reduces the β-catenin-mediated transcriptional activity as a result of competition with Tcf/Lef-1 transcription factors for β-catenin binding, inhibiting the expression of target genes associated with hair growth regulation [45,47,48]. Therefore, Wnt/β-catenin is considered as a potent target for promotion of hair growth by natural products. Recently, many natural compounds have been indicated to be inducers of Wnt/β-catenin signaling pathway. Oral administration of ginsenoside F2, a metabolite of ginsenoside-Rb1 by intestinal microorganism, has been shown to activate Wnt signal pathway through up-regulating expression of β-catenin and Lef-1, and down-regulating protein level of Dickkopf-1 (DKK-1), an antagonist of the Wnt/β-catenin pathway, thereby induction of the anagen phase and hair growth [49]. In addition, extracts from medicinal plants such as Polygonum multiflorum [50], Aconiti Ciliare Tuber [51], and Rumex japonicus Houtt [52] have been reported to induce anagen phase and stimulate hair growth through targeting the Wnt/β-catenin signaling pathway.

In addition, Shh/Gli pathway plays a crucial role in hair follicle morphogenesis during embryogenesis and regulation of follicular growth and cycling in the adult. Shh signaling is essential for the telogen-to-anagen transition, ingrowth of the epidermis, and consequent morphogenesis of hair follicle in vivo [39,53,54]. Shh mutant mice exhibit abnormal hair follicles containing a small dermal papilla and fail to undergo normal hair morphogenesis [54,55]. An antibody that blocks Shh activity is able to suppress hair growth in juvenile and adult mice, suggesting that Shh signaling is required for the anagen phase of the hair cycle [56]. Thus, we examined the effect of RGO on the expression of the Shh/Gli pathway-related protein in the TES-treated mice. As expected, RGO-treated mice exhibited significant increases in the expression levels of Shh/Gli pathway-related proteins including Shh, Smoothened (Smo), and Gli-1, which were suppressed by TES exposure (Figure 4B). Additionally, two major compounds in RGO including LA and SITOS were also found to activate the Shh/Gli signaling pathway in the TES-treated mice, although LA treatment did not produce an inductive effect on Smo protein expression. Whereas topical application of BICYCLO was unlikely to significantly accelerate protein levels of Shh and Gli-1, but likely to increase the Smo expression. Similarly, topical application of Polygonum multiflorum extract has been found to induce anagen phase in telogenic C57BL/N mice through up-regulating Shh expression [50].

Consequently, the expression of Wnt/β-catenin and the Shh/Gli signaling pathways-mediated target genes including Cyclins D1 and E were significantly up-regulated by treatment of RGO as well as its major components (Figure 4C). Cyclins D1 and E are believed to take part in controlling cell cycle and contributing to cell proliferation. β-catenin-deleted and Dkk1-overexpressing hair follicles exhibit decreased expression of cyclin D1, a direct Wnt/β-catenin target gene that promotes G1-to-S phase transition of cell cycle, possibly causing reduced hair follicle matrix cell proliferation [57]. These results demonstrates that RGO and its major components might induce premature anagen entry of telogenic hair follicles through the activation of Wnt/β-catenin and Shh/Gli-1 signaling pathways, thus promoting hair regeneration in the TES-induced delay of anagen entry in C57BL/6 mice.

## 3. Materials and Methods

### 3.1. Materials

Testosterone (>98%) and finasteride (>98%) were obtained from Tokyo Chemical Industry (Kita-ku, Tokyo, Japan). Linoleic acid and β-sitosterol were purchased from Sigma-Aldrich (St. Louis, MO, USA). Antibodies against β-catenin, Lef-1, TGF-β, Cyclin E, Smo, Shh, β-actin and peroxidase-conjugated antigoat were purchased from Santa Cruz Biotechnology (Santa Cruz, CA, USA). Cyclin D1, Bcl-2, Bax, and peroxidase-conjugated antirabbit antibodies were obtained from Cell Signaling Technology (Boston, MA, USA). Gli-1 antibody was purchased from Abcam (Cambridge, UK). BICYCLO was provided from the Laboratory of Synthetic Pharmaceutical Chemistry at Inje University (Gimhae, Korea). All other reagents used in this study were of the highest grade commercially available.

### 3.2. Preparation of Red Ginseng Oil (RGO)

RGO was prepared as described in the previous study [14]. Briefly, dried red ginseng powder with a particle size less than 400 μm was subjected to pilot-scale supercritical CO_2_ fluid extraction system (Ilshin Autoclave Co., Ltd., Daejeon, Korea). Extraction system was operated at 6500 psi and temperature at 65 °C. RGO was collected in a vial that was prefilled with a trapping solvent and chilled at 4 °C during the extraction process.

### 3.3. Animal Experimental Design

Male C57BL/6 mice (seven-weeks-old, 18–20 g) were purchased from Hyochang Science (Daegu, Korea) and maintained according to Institutional Animal Care and Use Guidelines of Inje University, who approved the mouse experiments with the approval number 2015-18 (Gimhae, Korea). Animals were housed in the plastic cages under a condition of humidity of 50 ± 5%, 12/12 h light-dark cycle with free access to standard food and water. After a week for adaption, dorsal skin of mice at 8 weeks of age, at which all of hair follicles were in the telogen phase, were shaved with a hair clipper and hair removal cream (Veet, Oxy Reckit Benckiser, Chartes, Frances). The mice were randomly divided into seven groups including group 1 (control group) which was the received vehicle; group 2 (TES group) was topically applied for 28 days with 0.5% TES that was prepared in 50% ethanol; groups 3–6 (sample groups) were daily treated with TES with 0.5% TES for 1 h and followed by topical treatment with 10% RGO, 1% LA, 1% SITOS, and 1% BICYCLO, respectively; group 7 (positive control) was topical applied 0.5% TES and then orally administered with 1 mg/kg finasteride (FINAS) that was prepared in 50% polyethylene glycol. The back skin of mice was photographed at day 0, 7, 14, 17, 21, and 28. Hair re-growth efficacy score as 0, 1, 2, 3, and 4 in correspondence to 0%, 0–20%, 20–40%, 40–60%, 60–80%, and 80–100% of hair growth was recorded. Dorsal skins of five mice from each group were collected after 17 and 28 days of depilation, snap-frozen in liquid nitrogen and stored in −80 °C for further experiments.

### 3.4. Histological Analysis

Dorsal skins from each group were fixed in 4% formaldehyde solution and embedded in paraffin to obtain longitudinal sections. The longitudinal sections were stained with hematoxylin and eosin (H&E) and digital photomicrographs were taken from representative areas using a digital camera (Paxcam, Villa Park, IA, USA). The number of hair follicles in anagen phase (A) and those in telogen phase (T) were counted, and A/T ration was determined.

### 3.5. Western Blot Analysis

Skin tissues were homogenized in cold RIPA buffer with protease inhibitor cocktail. Homogenates were centrifuged at 16,000× *g* for 10 min at 4 °C to obtain supernatants. To determine protein expression, an equal the mount proteins were separated by 10–12% SDS-PAGE and then transferred to polyvinylidene fluoride membrane using a semidry transfer system (Bio-Rad, Hercules, CA, USA). Membranes were incubated with the appropriate primary antibodies overnight and with horseradish peroxidase-conjugated secondary antibodies for 3 h. Finally, protein bands were visualized using enhanced chemiluminescence western blotting reagents (Santa Cruz Biotechnology, Santa Cruz, CA, USA).

## 4. Statistical Analysis

The data were expressed as mean ± SD values from three independent experiments. The statistical significance of difference between mean values was examined using analysis of variance followed by unpaired Student’s *t* test. *p* < 0.05 value was considered as a significant difference.

## 5. Conclusions

In the present study, we demonstrated that topical application of RGO promoted hair regeneration in the TES-induced delay of anagen entry in C57BL/6 mice, possibly contributed by major components of RGO including LA and SITOS. Treatment of RGO, LA, or SITOS resulted in premature telogen-to-anagen transition and increased the A/T ratio in TES-treated mice. In addition, RGO and its major components up-regulated the Bcl-2 level but down-regulated TGF-β expression in the TES-induced AGA models. Furthermore, inhibition of Wnt/β-catenin and Shh/Gli signaling pathways was restored in the presence of RGO, LA, or SITOS. These data suggest RGO may be used as a potent therapeutic agent for treatment of AGA and major components such as LA and SITOS synergistically produce hair re-growth activity of RGO.
